# A Word for Ourselves

**Published:** 1876-12

**Authors:** 


*TWENTY-THIRD YEAR OF*

### HALL'S JOURNAL OF HEALTH.

***E. H. Gibbs, M.D., Editor.***

VOL. XXIII.] DECEMBER, 1876. [NO. 12.

### *A WORD FOR OURSELVES.*

With this issue of the JOURNAL, its twenty-third year closes. For a period of two hundred and seventy-six months it has uttered its words of warning and of wisdom, on a vast range of topics of interest to humanity. It has striven earnestly in behalf of better modes of existence, more rational habits of life. That it has done a good work, in its own way, will not, we presume, be doubted.

Of the monthly issues of the JOURNAL, more than two hundred and forty were written by our late associate, DR. WILLIAM W. HALL, unassisted. He wielded a vigorous and rapid pen, and his earnest words were wont to carry conviction to the reader. His enthusiasm, his satire, and his humor, were alike contageous. Perhaps no writer on popular medical topics ever possessed an equally happy faculty for pointing the morals which he felt in duty bound to inculcate.

The "Centennial year" witnessed the departure of this friend of ours. He did not live to see the wonders of the great Exposition. He had been invited to be present at the opening, but professional duties detained him at home, and the same night he died, without a moment's warning.

Since DR. HALL's demise, the duties connected with the publication of his favorite JOURNAL, have chiefly devolved upon us. In each issue, however, some one or more articles, have appeared from his pen. He was in the habit of writing at odd moments, without reference to the immediate demand. By this means he accumulated a great mass of manuscript, which falls to us as his successor. These articles will appear from month to month until all have been printed in the JOURNAL. We shall thus have the benefit of our departed friend's vast stores of knowledge, even though he is no longer present with us.

For the rest, we have only to say that we shall do our best to make HALL'S JOURNAL OF HEALTH worthy of its good name, worthy the name of its illustrious founder. We have deemed it wise to lower its price, from and after this number, to one dollar and fifty cents per year. We do this not because we expect to lessen the value of the JOURNAL, but because printing and paper have lowered in price, and because we are convinced that the best way to secure the liberal accession of new subscribers, which we desire, is to make our price as moderate as possible. At a dollar and a half a year it makes a valuable and not very burdensome holiday gift, and one which any intelligent recipient would, we believe, feel very grateful for.

